# Factors Associated with Cigarette Smoking and Motivation to Quit among Street Food Sellers in Vietnam

**DOI:** 10.3390/ijerph15020223

**Published:** 2018-01-29

**Authors:** Xuan Thanh Thi Le, Lien Thi To, Huong Thi Le, Hanh Duc Hoang, Khanh Nam Do, Cuong Tat Nguyen, Bach Xuan Tran, Huyen Phuc Do, Luong Thanh Nguyen, Carl A. Latkin, Melvyn W. B. Zhang, Roger C. M. Ho

**Affiliations:** 1 Institute for Preventive Medicine and Public Health, Hanoi Medical University, Hanoi 100000, Vietnam; lethithanhxuan@hmu.edu.vn (X.T.T.L.); tolien.hsph@gmail.com (L.T.T.); lethihuong@hmu.edu.vn (H.T.L.); khanhdonam@gmail.com (K.N.D.); bach.jhu@gmail.com (B.X.T.); 2 Hanoi Department of Health, Hanoi 100000, Vietnam; hoangduchanh@hotmail.com; 3 Institute for Global Health Innovations, Duy Tan University, Da Nang 550000, Vietnam; cuong.ighi@gmail.com (C.T.N.); phuchuyen@gmail.com (H.P.D.); 4 Bloomberg School of Public Health, Johns Hopkin University, Baltimore, MD 21205, USA; carl.latkin@jhu.edu; 5 Vietnam Young Physicians’ Association, Hanoi 100000, Vietnam; 6 Biomedical Global Institute of Healthcare Research & Technology (BIGHEART), National University of Singapore, Singapore 117599, Singapore; ciezwm@nus.edu.sg; 7 Department of Psychological Medicine, Yong Loo Lin School of Medicine, National University of Singapore, Singapore 119228, Singapore; pcmrhcm@nus.edu.sg

**Keywords:** cigarette smoking, food sellers, motivated to quit, Vietnam, smoke-free at public places

## Abstract

Since 2013, smoke-free signs in public places, including in restaurants and food stores, have been introduced in Vietnam, aiming to prevent passive smoking. Although extensive research has been carried out on second-hand smoking among clients in public places (e.g., hospitals, restaurants) in Vietnam, no single study exists which captures the current practice of smoking among street food outlets. This study aims to estimate the prevalence of smoking and identify factors associated with smoking status and cessation motivation amongst food sellers in Vietnam. A cross-sectional study involving 1733 food providers at outlets was conducted in 29 districts in Hanoi capital, Vietnam, in 2015. The prevalence of smoking amongst food sellers was determined to be 8.5% (25% for men and 0.8% for women). The enforcement of the smoke-free policy remains modest, since only 7.9% observed outlets complied with the law, providing a room designated for smokers. Although approximately 80% of the participants were aware of the indoor smoke-free regulations in public places, such as restaurants and food stores, 40.2% of smokers reported no intention of quitting smoking. A percentage of 37.6% of current smokers reported that despite having intentions to quit, they did not receive any form of support for smoking cessation. Being male and having hazardous drinking habits and a poor quality of life were all factors that were significantly associated with smoking status. Additionally, having awareness of smoking’s adverse effects and being frequently supervised by the authority were associated with a greater motivation to quit. This study highlights the importance of an accompanying education and smoking cessation program in addition to the frequent inspection and reinforcement of smoke-free policy in food stores. This research extends on our knowledge of smoking prevalence and its factors related to smoking events and motivation to quit among street food outlets. Overall, this study strengthens the idea that more government efforts towards preventing passive smoking and smoking cessation education are necessary in restaurants and other street food outlets.

## 1. Introduction

Cigarette smoking is a global public health concern. Tobacco smoking is one of the leading causes of modifiable deaths worldwide [1]. Cigarette smoking in direct and indirect manners (passive smoking/second-hand smoking, SHS) also increases the risk of acquiring diseases such as tuberculosis, lower respiratory infections, cardiovascular disease, cancer, diabetes, and chronic respiratory disease [2]. The number of smoking-related mortalities is predicted to increase dramatically to 1 billion people in the 21st century [3]. Globally, 5% of all deaths are attributed to smoking, and 14% of adults over 30 years old died due to smoking-related diseases [4]. The prevalence of deaths related to smoking amongst South East Asian individuals has been found to be lower, with prevalence rates of 14% males and 5% females [5]. This is might be explained by a consequence of smoking prevalence, which has historically been lower than the global prevalence (32.1% versus 36.1% in 2012, respectively) and the prevalence in other regions, such as Europe (39%), the Western Pacific (48.5%), and the Eastern Mediterranean (36.2%) [6,7]. Notably, although trends in smoking have been declining globally, South-Eastern Asia, East Asia, and Eastern Europe remain the leading regions of smoking prevalence among men [6]. In Vietnam, smoking prevalence in the general population was 22.5% (45.3% among men and 1.1% among women) in 2015. Although the smoking prevalence declined by 5.3% during 2010–2015 [8], further efforts remain necessary toward becoming smoke-free in Vietnam and reducing the adverse effect caused by tobacco consumption.

In addition, tobacco use threatens global economic growth. According to the World Health Organization, 61% of tobacco-related burdens affect working-age people (between the ages of 15 and 59) [9]. Employees going out to smoke impose financial losses on their employers due to their absenteeism. In addition, low productivity rates are seen not only amongst the smokers, but also from the workers who are exposed to the environmental tobacco smoke (ETS) [10,11]. The tobacco-related healthcare cost surpasses the total tax revenues of the government that are collected from tobacco products [1]. Tobacco use also results in an economic burden in Vietnam due to the fact that the total costs of smoking were 24,679.9 billion VND (US $1173.2 million), accounting for 0.97% of national gross domestic product, in 2011 [12].

Although many countries have launched tobacco control provisions that restrict smoking in restaurants, recent reports suggest that SHS exposure varies dramatically by country, from 4.4% in Uruguay to 88.5% in China [13]. Prior studies have also highlighted the high prevalence rates of workers in restaurants and/or bars who use tobacco regularly or are exposed to second-hand smoke from the staff [14,15,16,17]. In addition, Lopez et al. suggested that people working in the food service industry were more likely to be exposed to smoking than in other fields, making it important to focus on this issue [18]. The other concern is that street food sellers commonly work in a group along the whole street. Thus, customers of street food outlets are also at high risk of second-hand exposure as smoking by one seller will affect other non-smokers.

In 2013, Vietnam enforced the Law on Prevention of Tobacco Harms, which banned smoking in indoor and outdoor public facilities, namely hospitals, educational facilities, and childcare facilities [19]. In other public places, including restaurants and food stores, smoking is banned indoors but permitted in a separated room for smokers [19]. The Decree 176/2013/ND-CP regulates that violators breaking the smoke-free provisions will be fined up to 300 thousand Vietnam Dong (approximately $15) [20]. The ban resulted from the fact that food sellers smoking at work might increase passive smoking exposure, leading to a 50% increase in lung cancer risk among food-service employees [21,22]. The recent global adult tobacco survey (GATS) in 2015 suggested that the passive smoking prevalence in Vietnam was 80.7% at restaurants, 53.5% at home, 36.8% at the workplace, 18.5% on public transport, and 16% in educational facilities [23]. Additionally, the rationale of this ban was based on some evidence that people in indoor public places where smoking remains prevalent were exposed to 8.9 times higher PM_2.5_ (fine particles in the air 2.5 µm in size) levels than in places where smoking is completely prohibited [24].

However, the enforcement of this ban in street food service facilities across Vietnam is faced with many challenges. Food purchasing facilities are often narrow with limited space availability, and as such it is challenging to design a separated room or outdoor area for smokers. Additionally, research to date has focused on smoking exposure in restaurants, where businesses tend to be well-organized and workers are constantly supervised. This is, however, not the case in street food service facilities, as they are often unregulated and there remain challenges in enforcement. A survey by the Health Bridge Canada in Vietnam found that only 49/195 restaurants had a “no smoking” sign presented, and 9 restaurants had a separated outdoor areas for smokers [25]. One major drawback of this survey is that it did not include the street food service facilities, which often provide food services for many clients, including vulnerable populations such as children and elderly people. There remains a paucity of current evidence to date on the implementation of the smoke-free policy among street food diners. Additionally, there is a growing body of literature that recognizes factors related to smoking, including gender, age, education [8], knowledge on smoking harms [26], health status, and an absence of a smoking ban [27]. However, these studies did not explore characteristics and risk factors amongst street food vendors.

The main purpose of this study is to determine the prevalence of smoking and identify factors associated with smoking status and motivation to quit amongst sellers at food service facilities in Hanoi, Vietnam. This study provides an exciting opportunity to advance our knowledge of anti-smoking strategies to strengthen smoke-free policy in public places as well as promote a better life for both food-service workers and customers. The findings will also make an important contribution to investigating the factors that are associated with motivating oneself to quit, which will have valuable implications for cessation services among street stores in Vietnam.

## 2. Materials and Methods

### 2.1. Study Setting and Population

A cross-sectional structured interview study with people working in the street food industry was conducted in October 2015 among 176 communities in 29 urban and rural districts of Hanoi, the capital of Vietnam.

Normally, in Vietnam’s street food industry, the same workers are responsible for processing and selling food. Food sellers responsible for contact with customers were the main target of the interview, but if no food seller was available for interview, a processor who was in charge of cooking was selected to do the interview instead.

The sampling frame of 20,600 food service facilities was obtained by the district authorities. Food service facilities were categorized as follows:

- Street food diners: have a dining area where customers can sit and eat food; and
- Street food vendors: provide cooked food items for taking away only.

A multi-stage sample design was employed: in each ward/community, ten food service facilities were sampled and one worker was interviewed from each facility. Food service facilities were included in the sample if all of the following inclusion criteria were met:

- a food seller or processor aged at least 18 years old was available for interview; and
- the food serve facility was currently selling food items at the interviewing time.

A total of 1570 food sellers and 180 processors were recruited from 176 communities in 29 urban and rural districts. This corresponds to 8.5% of food service facilities in the sampling frame. Participants were excluded if they had any health problems that impaired their communication during the interviewing time. No variable has >5% missing data, or failed to give consent. After exclusions, 1733 participants were included in the study (99% of the original sample).

### 2.2. Study Variables

**Socio-demographic status**: Variables of interest included age, gender, marital status, religion, ethnicity, and educational attainment using the question: “*What is your highest level of education obtained?*”.

**Smoking-related characteristics**: In this study, we assessed current smoking status by using a questionnaire: “*Are you currently smoking in the last 30 days?*” and “*How many cigarettes did you smoke per day within the last month?*”. Respondents were labeled as a current smoker if their response was “Yes” to the first question.

**Motivation to quit:** To evaluate a participant’s motivation to quit smoking, the transtheoretical model (TTM) was used to identify the sequential stages of change for smoking cessation. Five stages were identified, including: (i) Pre-contemplation: no intention to stop; (ii) Contemplation: motivation to stop in <6 months; (iii) Preparation: intend to stop in <1 month; (iv) Action: have actively quit and are maintaining early abstinence; and (v) Maintenance: currently abstinent [28,29]. The question “*Are you considering smoking cessation?*” was followed by four options: “*No thought of stopping*”, “*I should stop but am not quite ready*”, “*I am beginning to think about smoking cessation*”, and “*I have already taken action to stop*” that corresponded to the stages of (i) → (iv). Respondents who reported abstinence from smoking for more than 6 months were considered to be in stage (v). Participants in the contemplation or preparation action stages were considered to be “motivated to quit”. Additionally, smokers were asked about the primary support for smoking cessation or information about this they had received previously.

**Health-related quality of life (HRQOL)**: The EuroQol includes five domains and five grades (EQ-5D-5L) that were utilized to measure “health-related quality of life”, including (i) mobility; (ii) self-care; (iii) usual activities; (iv) pain/discomfort; and (v) anxiety/depression with five grades of response: no problems, slight difficulties, moderate difficulties, severe difficulties, and extreme difficulties. Responses regarding the five domains were generated from 1733 participants and then transformed to an individual index (the EQ-5D index) utilizing the temporary scoring from Thailand’s value package [30,31]. This individual score ranges from 0 to 1, with 1 for no problems in any of the five domains and 0 for problems in all five domains. In addition, the EuroQol-Visual analogue scales (EQ-VAS) assesses the self-rated health of subjects on a vertical scale with scores ranging from 0 to 100 points, classified ‘the worst health in your rating’ and ‘the best health in your rating’, respectively.

**Alcohol use**: To measure alcohol use, a brief version of the alcohol use disorders identification test-consumption (AUDIT-C) instrument was employed [32]. The Vietnamese version has been validated elsewhere [33,34,35]. This instrument comprised three questions, including “*How long have you drunk in the last 6 months?*”; “*How many cups (4–5 mL) do you drink on one occasion?*”; and “*How long have you drunk more than six cups per occasion?*”. The total score ranges from 0 to 12. The higher scores indicate a higher level of alcohol abuse. The threshold of 4 or higher for men and 3 or higher for women was used to identify “hazardous drinkers”. Respondents who have reported a positive response to the question of drinking more than six cups per occasion were labeled “binge drinkers”. 

**Other predictors of smoking status:** Factors related to smoking consisted of knowledge of the adverse effects of tobacco, awareness of the smoking ban in food service facilities, frequency of tobacco advertising and promotion, frequency of authority’s inspection, having a separated area for smokers, and the frequency of tobacco being provided in stores. Frequency of audits by inspectors and tobacco advertising/promotion were classified in three levels (Never/rarely: annually, occasionally: quarterly, often: monthly). Participants were defined to have knowledge of the smoking ban if they were aware of the indoor smoking ban in food stores with a separated room for smokers. Those who mentioned at least five smoking-related illnesses (e.g., lung diseases, cancers, chronic obstructive pulmonary disease, heart disease, birth defects and miscarriage, etc.) were defined as having knowledge of smoking harms.

### 2.3. Data Analysis

The data were analyzed using STATA software version 13.0 (Stata Corp. LP, College Station, TX, USA). *T*-test and Chi-square tests were introduced to measure the differences among socio-economic structure, alcohol status, and health-related variables, stratified by current smoking status (yes/no). A *p*-value < 0.05 was considered statistically significant.

To identify factors associated with smoking status and motivation to quit, a multivariate logistic regression method was applied with the incorporation of covariates, such as participants’ perception, HRQOL, alcohol status, demographic variables, and frequency of authority inspection. Firstly, a forward stepwise approach was used to identify factors associated with smoking status among food sellers by importing all covariates into the model. Secondly, only factors significantly associated with the primary outcome of a bivariate analysis at *p* < 0.25 were presented in the multivariate logistic model manually. The final model considered statistically significant factors at a *p*-value < 0.05.

### 2.4. Ethical Consideration

The ethical clearance of the study protocol was granted by the Institutional Review Board of Hanoi Department of Health, based on the guidelines of the Vietnam Ministry of Health (code: 06/CCATVSTPHN). All participants were provided with an informed consent participation sheet as well as a participant information leaflet. First, the purpose of this study was explained to a prospective participant, and written informed consent was obtained once the participant agreed to do the interview. Participants could withdraw at any time and were informed that their responses would be kept confidential.

## 3. Results

Table 1 shows the sample characteristics. Among the 1733 participants, the mean age was 41.5 (SD = 10.9) years. Most of them were females (68.0%), living with a spouse (89.5%), religiously identified as a cult of ancestor (90.6%), and mainly obtained a high school degree (54.8%). Street food vendors were the most dominant (59.5%), followed by street food diners (40.5%). Most of the stores had a small size to serve less than eight tables of clients (69.9%), and few stores had separated room for smokers (7.9%). Although approximately 80% of participants were aware of the harmful effects of smoking and were aware of the smoking ban in food service facilities, only 7.9% of food sellers worked at facilities that had a designated room for smokers, complying with the smoke-free provisions in public places. The mean EQ-5D and EQ-VAS of food sellers was 0.94 (SD 0.1) and 92.4 (SD 8.3), respectively (Table 1).

Table 2 presents that smoking prevalence was 8.5% overall for the participants, with 25% for men and 0.8% for women, with this difference between the two groups being statistically significant (*p* < 0.01). The smoking proportion was also significantly different by marital status, outlet categories and size of food stores, and age (*p* < 0.05).

Among participants currently having health problems, the proportion of smoking sellers in the physical pain group was 5.4%, the proportion in the low mood group was 6.1%, and the proportion in the group having difficulties with daily activities was 8.7%. The difference of smoking prevalence rates between participants who had illness and participants who had no illness was not statistically significant (*p* > 0.05). Approximately 8.8% of respondents in the group having chronic illness within 3 months were smokers. The prevalence of smoking was statistically significantly different between binge/hazard drinkers and those who were not binge/hazard drinkers (*p* < 0.01). The proportion of smokers being binge drinkers and hazardous drinkers was 63.7% and 59.5%, respectively. Among those having perception of the adverse effect of smoking and the smoke-free policy in food outlets, the smoking proportions were similar (approximately 9%, *p* > 0.05) (Table 2).

The proportion of motivation to quit is also shown in Table 2. Amongst current smokers, the proportion of participants who intended to quit smoking was 59.8%. The difference in motivation to quit was statistically significant between food stores and street food vendors, with factors including the frequency of authority’s inspection, the frequency of tobacco advertising, and the promotion at food outlets, and knowing that smoking is banned in food stores (Table 2). Of the 144 current smokers, the proportion of motivation to quit was highest among those receiving quit advice from acquaintances and health staff (approximately 70%), while only 41.7% who have never received information about quitting were interested in smoking cessation (*p* = 0.001).

Results from the multivariate logistic regression show that men (odds ratio (OR) = 50.7; 95% CI = 21.6–119.2) and hazardous drinkers (OR = 3.5; 95% CI = 2.2–5.6) were more likely to be smokers. Participants with greater EQ-VAS scores were less likely to be current smokers (OR = 0.96; 95% CI = 0.94–0.99). Sellers who reported chronic illness tended to be smokers than others; however, this association was not statistically significant. In addition, participants who were aware of the adverse effects of smoking had an 8 times statistically greater motivation to quit than those who were not (OR = 8.7, 95% CI 1.1–68.9, *p* < 0.05). Interestingly, sellers at food outlets who were rarely supervised by authority and ongoing tobacco sellers were less likely to be motivated to quit (OR = 0.23, 95% CI 0.08–0.61, *p* < 0.01 and OR = 0.04, 95% CI 0.01–0.38, *p* < 0.01, respectively). Sellers at food stores have approximately a three times greater motivation to quit than street vendors with a borderline significant trend (*p* = 0.07) (Table 3).

## 4. Discussion

This is one of the first studies to explore tobacco consumption amongst sellers in the food service industry and the factors associated with smoking and motivation to quit in Vietnam. This study found that the prevalence of tobacco use among sellers at food service facilities in our sample was 8.5% (25% for men, significantly higher than in women (0.8%)). Although approximately 80% of the participants were aware of the indoor smoke-free regulations in public places, such as restaurants and food stores, only 7.9% of observed outlets reported having a separated area designed for smokers, which revealed the modest enforcement of the smoke-free policy. In addition, 59.8% of smokers were motivated to quit smoking. Being male, having hazardous drinking habits, and having a poor quality of life were significantly associated with smoking status. Additionally, having awareness of smoking’s adverse effects and frequently being inspected by the authority were associated with a greater motivation to quit.

The overall rate of smoking in our study was much lower in comparison with the general population in Vietnam (22.5% for overall, 45.3% for men, and 1.1% for women) [8] and handlers and managers at food service facilities in Riyadh, city of Saudi Arabia (26%) [36,37]. The smoking prevalence difference by gender among food sellers was also in line with the Vietnamese population as a consequence of a social norm in Asian society which discourages smoking among women. The occurrence of tobacco use among food sellers in this study was even lower than the age-standardized prevalence of current tobacco smoking among Vietnamese young adults, aged from 15 years old via an online survey in 2017 (10%) [38], or adults in a rural area (16.1%) [39]. The reason might come from the fact that most of the street food sellers were female (68%), which reflects the current labor market of the food industry in Vietnam, where women are the dominant staff with very low smoking prevalence (0.8%). Additionally, due to resource scarcity, interviewing the one person in charge of selling food in one outlet has limited our chance to detect the smoking prevalence amongst coworkers. Another bias might come from the self-assessed smoking status without biochemical validation. Thus, the smoking prevalence might be underestimated compared to the actual rate in this population.

Even though most of the participants (80%) were aware of the dangers of smoking as well as the fact that restaurants were designated to be a smoke-free environment, this proportion was lower than proportion of participants knowing this law in the general population retrieved from the Global Adult Tobacco Survey 2015 (95.9%) [23]. Additionally, approximately 37.6% of smokers had not ever received any support or information to cease smoking. The majority of smoking respondents had a tendency to receive support from relatives/friends (more than 35.2%) and health staff (roughly 18.4%). This trend may be due to the low coverage of smoking cessation services as well as the counseling quit-line in Vietnam [40]. Another reason for high smoking prevalence is that tobacco products are available and easily accessible with a low price in Vietnam (around 1 US$ per pack of 20 sticks) [41]. Therefore, increasing tobacco taxes is one of the most effective ways to reduce smoking prevalence, particularly among disadvantaged groups [42].

This study also suggested that smokers being supervised frequently by an authority’s inspection and smokers who had perception of the harmful effect of smoking were more likely to be motivated to quit than those who did not. These findings highlight the role of the authority for inspection, the reinforcement of the frequent inspection, and monitoring for compliance with smoke-free provisions at street food sellers, since a financial sanction will be applied for violators breaking the law [19] and only 7.9% stores had separated room for smokers. Additionally, scaling up anti-smoking education and communication campaigns to provide proper knowledge of smoking’s detrimental effects based on the street food service facilities in Hanoi is necessary and beneficial for both active and passive smokers [43,44]. Introducing quit-line support widely and providing consulting services is also a promising approach to help smokers be ready to materialize their intention to quit and remain a prolonged abstinent. Family and friends might be engaged to support smokers, as their influence might result in a positive effect for nicotine dependents. Finally, for the well-being of both staff and customers, especially vulnerable subjects to becoming addicted such as adolescents and former smokers, it is important to move them along the chain of smoking cessation stages (from the contemplation and preparation stages to the action stage) toward smoking cessation in the long-term.

The most obvious finding to emerge from the analysis is that people having a lower self-rated health score (EQ-VAS) were more likely to smoke a cigarette. Our finding was consistent with a previous study in the United States, which indicated that the greatest smoking prevalence among workers in food services were observed among those having poor health with no health insurance [45], and a low quality of life and depression were related to smoking initiation [46]. This result could be explained that people perceive smoking to be helpful in the reduction of stress, depression [47], or chronic pain [48]. However, convergent findings from both prospective researchers and cross-sectional researchers suggest a bidirectional relationship between depression and smoking, as smoking might increase the risk for subsequent depression development [49]. Furthermore, respondents who were hazardous drinkers were also more likely to smoke (OR = 3.5). It might be due to their interest or habit, negative moods, the influence of peer colleagues, friends, parents, or stress [50] and reflect the cultural context in Vietnam with tobacco and alcohol comorbidity, especially among male young adults [51,52]. Our finding found a variety of risk behaviors among food sellers as well. Therefore, health education campaigns or substance abuse treatment, tailoring for smokers with alcohol abuse as well, should be provided for seekers of a healthy lifestyle among food sellers.

This study’s strength consisted of a large sample size (1733 street food service facilities) and a high retention rate (99%), the incorporation of various validated scales (e.g., AUDIT-C, EQ-5D-5L, and EQ-VAS). However, several limitations should be acknowledged. First, the cross-sectional method does not allow for assessing how the smoking prevalence changes over the time or the causal relationship, especially, to evaluate the effect of the smoke-free policy at food outlets. Secondly, the self-reported assessment of smoking status might introduce recall bias, including social-desirability bias, which might have produced an underestimation of the true smoking rate in this population. Thirdly, participants were selected from a sampling frame obtained by the district authority, which might not capture all current street food vendors, especially those engaged in the food industry by season or those who are temporary. Finally, a generalization of these findings should be applied with caution, as data were collected only in Hanoi, an urban city of the northern area in Vietnam.

## 5. Conclusions

Smoking in food service facilities and the associated secondhand smoking exposure is harmful to the well-being of sellers, staff, and customers. Smoking status among food sellers was associated with alcohol abuse, while the motivation to quit was highly correlated to the perception of smoking’s harmful effects and being frequently inspected by authority. Apart from the reinforcement of smoke-free provisions at public places, tobacco control education and smoking cessation services are necessary to reduce cigarette smoking in restaurants and food shops in Hanoi.

## Figures and Tables

**Table 1:** Sample characteristics among street food service facilities, Hanoi, Vietnam (*n* = 1733).

Characteristics	Total Sample	
	*n*	%
Gender		
Male	552	32.0
Female	1175	68.0
Marital status		
Single	157	9.1
Living with spouse/partner	1543	89.5
Divorced/widowed	24	1.4
Religion		
Buddhism	114	6.6
Catholicism	37	2.1
Cult of ancestor	1565	90.6
Others	12	0.7
Ethnic		
Kinh	1720	99.7
Other	5	0.3
Education attainment		
Less than high school	501	29.1
High school	944	54.8
More than high school	277	16.1
Type of food outlet		
Street food diners	697	40.5
Street food vendors	1023	59.5
Size of food stores		
Small: <8 tables	1204	69.8
Medium: 9–30 tables	488	28.3
Large: ≥30 tables	33	1.9
Knows that tobacco causes harm	1450	87.6
Knows that smoking is prohibited in food stores	1390	80.2
Works at a food outlet that has an area designed for smokers	137	7.9
Age, Mean year (SD)	41.5	10.9
Self-rated health (EQ-VAS), Mean (SD)	0.94	0.1
Health-related quality of life (HRQOL), Mean (SD)	92.4	8.3

**Table 2:** Prevalence of smoking and prevalence of motivation to quit by factors among food sellers.

Factors	Smoking Prevalence (*n* = 1733)		Prevalence of Motivation to Quit (*n* = 144)	
**Prevalence**	***n* (%)**	***p*-value**	***n* (%)**	***p*-value**
Total sample	148 (8.5)		86 (59.8)	
Gender				
Male	138 (25)	<0.01	84 (60.9)	0.18
Female	9 (0.8)		2 (33.3)	
**Participant related factors**	***n* (%)**	***p*-value**	***n* (%)**	***p*-value**
Marital status				
Single	22 (14.1)	0.01	16 (76.2)	0.09
Living with spouse/partner	125 (8.1)		70 (58.3)	
Education attainment				
Less than high school	34 (6.8)	0.27	24 (68.6)	0.57
High school	86 (9.1)		52 (59.7)	
More than high school	26 (9.4)		14 (56)	
Health problems, (Yes versus No)				
Having Pain/Discomfort	3 (5.4)	0.39	4 (75)	0.54
Having Anxiety/Depression	6 (6.1)	0.37	4 (57.1)	0.83
Having difficulties in mobility	0 (0)	0.12	0 (0)	N/A
Having difficulties in self-care	0 (0)	0.14	0 (0)	N/A
Having difficulties in usual activities	31 (8.7)	0.86	13 (39.4)	0.01
Having chronic illness within 3 months (Yes versus No)	5 (8.8)	0.86	2 (40)	0.36
Binge drinking (Yes versus No)	123 (63.7)	<0.01	39 (56.5)	0.34
Hazard drinking (Yes versus No)	88 (59.5)	<0.01	34 (56.7)	0.39
Knowing that tobacco causes harm (Yes versus No)	125 (8.7)	0.91	81 (64.3)	0.07
Knowing that smoking is banned in food stores (Yes versus No)	124 (9.0)	0.18	82 (64.1)	0.04
Main support for/method of quitting smoking have been used previously				
Never received anything			20 (41.7)	0.001
Quit advice from health staff			17 (70.8)	
Quit advice from relatives/friends			36 (78.3)	
Ever used NRT			1 (100)	
Self-quit method			9 (90)	
**Outlet related factors**	***n* (%)**	***p*-value**	***n* (%)**	***p*-value**
Type of food outlet (*n* = 1708)				
Street food diners	71 (10.2)	0.09	42 (57.5)	0.43
Street food vendors	73 (7.2)		44 (63.9)	
Size of food stores				
Small: <8 tables	87 (7.3)	0.01	50 (60.5)	0.87
Medium: 9–30 tables	51 (10.6)		33 (62.3)	
Large: ≥30 tables	27 (18.2)		3 (50)	
Works at a food outlet that has an area designed for smokers (Yes versus No)	11 (8.2)	0.85	8 (72.7)	0.39
Being tobacco point of sale (Yes versus No)	25 (15.4)	<0.01	18 (66.7)	0.55
Frequency of tobacco advertising, and promotion at food outlets				
Never/Rarely (annually)	9 (13.2)	0.29	1 (12.5)	0.01
Occasionally (quarterly)	13 (9.6)		13 (76.5)	
Often (monthly)	123 (8.1)		75 (61.9)	
Frequency of authority’s inspection				
Never/Rarely (annually)	93 (9.2)	0.45	48 (52.2)	0.01
Occasionally (quarterly)	23 (7.1)		18 (72)	
Often (monthly)	30 (7.9)		24 (80)	

**Table 3:** Factors associated with current smoking status and motivation to quit.

Model	Odds Ratio (OR) (95% CI)	*p*-Value
**Factors associated with smoking status (*n* = 1706)**		
Gender (Male versus Female)	50.7 (21.6–119.2)	<0.01
Education attainment (>high school versus <high school)	0.7 (0.4–1.2)	0.20
Hazard drinking (Yes versus No)	3.5 (2.2–5.6)	<0.01
Having chronic illness (Yes versus No)	3.5 (0.95–12.9)	0.06
Self-rated health (EQ-VAS)	0.96 (0.94–0.99)	<0.01
**Factors associated with motivation to quit (*n* = 136)**		
Self-rated health (EQ-VAS)	1.07 (1.01–1.16)	<0.05
Having difficulties in usual care (Yes versus No)	0.12 (0.02–0.59)	<0.01
Being supervised by inspectors (Low versus High frequency)	0.23 (0.08–0.61)	<0.01
Being tobacco outlets as well (Yes versus No)	0.04 (0.01–0.38)	<0.01
Type of food outlets (Street food diners versus Street vendors)	2.9 (0.91–9.6)	0.07
Knowing that tobacco has harms (Yes verus No)	8.7 (1.1–68.9)	<0.05
